# Perception, willingness, and practices of telemedicine in patients with chronic diseases: implication of digital health in patients' perspective at a tertiary care hospital in Ethiopia

**DOI:** 10.3389/fpubh.2023.1234436

**Published:** 2023-08-07

**Authors:** Eyayaw Ashete Belachew, Demis Getachew, Adeladlew Kassie Netere, Eshetie Gizachew, Ashenafi Kibret Sendekie

**Affiliations:** ^1^Department of Clinical Pharmacy, School of Pharmacy, College of Medicine and Health Sciences, University of Gondar, Gondar, Ethiopia; ^2^Department of Pharmacology, School of Pharmacy, College of Medicine and Health Sciences, University of Gondar, Gondar, Ethiopia; ^3^Department of Information System, College of Informatics, University of Gondar, Gondar, Ethiopia

**Keywords:** attitude, chronic diseases, perception, practice, telemedicine, willingness

## Abstract

**Background:**

Technology-based healthcare services have important implications for the diagnosis, prevention, and treatment of diseases, as well as providing access to high-quality care that both the patient and the healthcare practitioner can benefit from. To access medical information, patients have also searched for methods of technology-based healthcare services like telemedicine (TM). However, little is known regarding the perceptions, willingness, and practices of TM among Ethiopian patients, especially in the study setting.

**Objective:**

This study assessed the perceptions, willingness, and practice of TM among patients with chronic disease at the University of Gondar Comprehensive Specialized Hospital (UoGCSH), Northwest Ethiopia.

**Methods:**

A cross-sectional study was conducted from June 1 to July 30, 2022, among patients with chronic diseases who were on follow-up at the UoGCSH. Eligible participants were included in the study using a systematic random sampling technique. A structured questionnaire was used and recorded in the Kobo data collection tool. The collected data were managed and analyzed using the Statistical Package for Social Science (SPSS) version 26.

**Results:**

Out of 422 patients approached, 384 (91% response rate) were included in the final analysis. The mean (±SD) age of the participants was 48.07 ± 16.17 years. The overall perceptions mean (±SD) score of the respondents was 3.92 ± 1.06. Generally, near to three-fourths (71.1%) of the participants had a positive perception of TM services, and around two-thirds (63.3%) had a willingness to be involved in the TM service. However, only around one-fourth (24.5%) of the participants were perceived to have a high level of TM practice currently.

**Conclusion:**

The findings suggest that although the level of perception and willingness of TM services among patients with chronic diseases was positive, their level of practice was low. Therefore, creating awareness and suitable conditions to improve their utilization of TM could be important.

## Introduction

Digital-based intervention has been popular in the self-management of patients with chronic diseases, particularly during the COVID-19 pandemic, when patients faced difficulties accessing face-to-face health services with healthcare providers (1–4). Telemedicine (TM) has been used for communication between physicians, nurses, paramedical personnel, and patients in need of medical care (5, 6). The origin of TM, that is, the support of health care services through communication and technology, can be traced back to the time during which electronic devices were invented (7). TM has been used to describe the direct provision of healthcare services, and its applications were demonstrated as teleconsultation, remote psychotherapy, remote imaging, telepathology, tele dermatology, patient care at the first level of health care, specialized counseling systems, remote surgery, remote anesthesiology, remote cardiology, remote radiology, remote oncology, home monitoring, patient education, continuing medical education, and remote home medical care (8, 9). Globally, different studies were conducted to show the adoption, use, and utility of TM during COVID-19 (10–13).

Chronic diseases have a significant impact on people's lives in the following ways: impaired mobility, reduced quality of life, negative emotions, increased economic burden, and a higher mortality rate. Despite this devastating potential, the incidence of chronic diseases is increasing (14, 15). According to the World Health Organization's 2015 report, chronic diseases killed 38 million people each year and have become a public health concern (15). Deaths account for roughly 80% of all deaths in low- and middle-income countries. Morbidity and mortality from chronic diseases are increasing faster in sub-Saharan Africa than at any other time in history. Ethiopia is no exception to the burden imposed by such chronic diseases (16). The prevalence of chronic non-communicable disease (NCD) in Ethiopia has ranged from 29 to 35%: diabetes at 5%, cardiovascular disorders at 13.4%, and respiratory conditions at 1–18% (1–18%) (17). Aside from its high prevalence, chronic disease management is difficult because it necessitates long-term follow-up by health care providers and patients, as well as a large amount of resources. This results in a scarcity of health-care resources available to patients and their families and serves as a driving force for telehealth innovation (18). TM enables remote monitoring of vital signs in patients with chronic conditions, and by doing so, it decreases mortality and hospitalization while increasing quality of life (4, 19–21). It eliminates the need to go to the doctor's office and sit side by side with others, which could lead to the transmission of communicable diseases (22). It also reduces rates of exacerbation and hospitalization in patients with asthma and chronic obstructive pulmonary disease (COPD) by allowing patients to receive better medical attention at the convenience of both the doctor and themselves (22). A review also disclosed that the use of digital-based messages helps in improving hypertension, diabetes and asthma (23).

Although interest in telehealth is growing, its implementation is not common practice yet. Initiatives for e-health applications are not similar and consistent throughout the world. For example, in the Netherlands, the implementation of e-health is developed through the collaboration of caregivers, caretakers, and health insurance (14). People living in low-income countries, such as African countries, face more health problems than those in developed countries, and they have limited access to health innovations (24). When it comes to Ethiopia, which is the second-most populous country in Africa, with over 117 million people in 2021, in order to meet the healthcare needs of such a large population, the implementation of TM is recommended to expand (25). Ethiopia's Ministry of Health (MoH) recently launched a digital and innovation center where experts can synthesize, promote best practices, and scale up innovation tools. According to the April 30th, 2021 report, ~54.7 million people were telecommunications subscribers. There were ~54.7 million mobile voice subscribers, 25 million internet subscribers, 349,000 fixed broadband subscribers, and 923 fixed service subscribers. The telecom coverage was reported to be 85.4–95%, and the density was 50% (25).

Knowing the global and national implications of chronic disease management, developed countries advocated for a new technology, TM that supports the health system. It aids in lowering health-care costs, increasing access to care, reducing the need for a larger workforce, and reducing time waste. Although its implementation is in its early stages, Ethiopia needs to implement TM after learning about its benefits. However, to the best of authors knowledge and broader search, assessment of patients with chronic diseases perceptions, willingness, and practice of TM has not been explored yet. Even though various literatures are conducted during COVID-19 world concerning to the use of TM and its implication, there is scarcity of information in the study area.

Therefore, the main research questions are: (I) Do patients with chronic diseases perceive TM as good or bad? (II) Are patients with chronic diseases willing to use TM or not? (III) Do patients practice TM or not? To answer these research questions, this study assessed perceptions, willingness, and practice of TM among patients with chronic diseases at the UoGCSH, Northwest Ethiopia. The findings will serve as a baseline for identifying opportunities and barriers from the patients' perspective to ensure the delivery of better healthcare through the implementation of TM.

## Methods and materials

### Study design and setting

An institutional-based cross-sectional study with patient interviews and medical record review was carried out at UOGCSH ambulatory chronic care from June 20 to July 20, 2022. The UoGCSH is a comprehensive public referral health facility in northern Ethiopia that serves as a teaching hospital for students from the University of Gondar. The hospital is ~738 km from Ethiopia's capital city. The UoGCSH statistics and information office revealed that the hospital's ambulatory care follows up with patients every Monday to Friday and serves 15,000 patients with chronic conditions such as HTN, diabetes, asthma, COPD, heart failure, epilepsy, and other cardiovascular disorders as of the November 2019 report.

### Study participants

All patients with common chronic diseases who had been followed for their medical condition at the ambulatory chronic care unit of the UoGCSH during the study period were included as study participants. To be included, they could be adults (age higher and equal to 18), diagnosed and followed for at least one chronic disease, and agreed to participate in the study. Patients who were severely ill and unable to provide an interview, those who were with sever neurological and psychiatric problems, and patients with hearing problems were all excluded.

### Sample size and sampling technique

The sample size was calculated using the single mean population proportion formula with a 95% confidence level (Z = 1.96), a proportion of the outcome (i.e., perceptions, willingness, and practice of TM) of 50% (P = 0.5) to obtain an adequate sample size and a relative precision of 5% (D = 0.05).


$$\begin{array}{l} n=\frac{{\left(\frac{Z\partial }{2}\right)}^{2}P\left(1-P\right)}{D^{2}}=\frac{{\left(1.96\right)}^{2}*0.5*0.5}{{\left(0.05\right)}^{2}} \end{array}$$

*n* = 384 .16≈ 384

Where; Zα/2 = confidence level at 95% (standard value of 1.96); P = estimated prevalence or proportions of the outcome; d = range of CI or margin of error.

Finally, considering a 10% contingency for possible non-response, the calculated sample size was to be 422.

A systematic random sampling method was used to include study participants. The total number of chronic patients who visit this hospital per month is about 15,000; from this, 1,500 patients who fulfilled the inclusion criteria visited the hospital every month, and 300 patients who fulfilled the inclusion criteria were found to have an appointment during the data collection period. Thus, we had 1,800 visitors in a month (taking into consideration that the sample was collected within a month). As a result, the sampling fraction (k-interval) is 1,800/422 = 4 (approximately). As a result, the first participant in the study was chosen at random, and each subsequent participant was chosen every four persons. The study subjects who met the inclusion criteria were considered, and if one was deemed ineligible, the next one was chosen, and the same approach was used throughout the data collection procedure.

### Data collection tools and techniques

Data on patients' perceptions, willingness, and practice of TM were collected using validated data collection instruments. The data were collected following the adoption of questionnaires from various studies (14, 22) and were prepared using the Kobo data collection tool. The data collection format is divided into four sections. Part I: focusing on the study population's socio-demographic characteristics (age and gender, marital status, educational level, residence, work status, internet or mobile phone usage status, religion, mode of transportation, and health service coverage) and clinical characteristics questionnaires (type of chronic disease, duration of disease, frequency of visit, perception of waiting for health service, and e-health usage were assessed using structured questionnaires); Part II: Perception and/or opinion toward TM (in this part, patients' past and present perceptions and opinions about its benefits in terms of cost reduction, time saving, quick access, and access to remote areas were assessed). This perception toward TM use was measured by 14 questions on a five-level Likert (5 = strongly agree, 4 =agree, 3 = neutral, 2 = disagree, 1 = strongly disagree) scale questionnaire, and the overall level of perception was determined and categorized by using the overall mean score of the questionnaires. Participants with scores above the mean have a positive perception of TM, while those with scores below the mean have a negative perception of TM. Part III was composed of patients present as well as their future willingness to use TM. The questionnaire consists of 16 questions: three questions with a score of 4 (0–3), one question with a score of 3 (1–3), and 12 yes or no questions (1 = yes and 0 = no). Therefore, the overall score of the questionnaire was 24 points. To rate the overall willingness of the participants to engage in TM, we have categorized those scores ≥ 13 as having good willingness and those scores below 18 as having poor willingness to engage in TM. Part IV: Focuses on their current practice in the TM (their rate or frequency of usage and the type of information they extract from an e-health service). To measure the practice of respondents, we developed 12 yes-or-no questions. The overall level of practice was determined by the score of the questions. Those participants who answered yes to questions six and above were considered to have good practice of TM.

Cronbach's alpha was employed to determine the internal consistency of the data collection tools. Perception toward TM (α = 0.78), thoughts to update their information toward TM (α = 0.82), willingness to use TM (α = 0.79), practice of TM (α = 0.83), and frequency of using TM (α = 0.88) all indicated that the tools have an acceptable range of reliability.

### Data quality control technique

Before collecting data, the literature on the questionnaires was reviewed. A pretest was conducted on 21 patients with chronic diseases (5% of the samples) having follow-ups at Debark general hospital. Then, after the pretest feedback, the actual data was collected. Following data collection, proper categorization and coding of the data were performed, and the collected data were reviewed for completeness and accuracy by checking the recorded data. After entering the data into SPSS, it was double-checked for accuracy.

### Data entry, management, and analysis

Data was collected by the Kobo data collection tool and then downloaded to XLS and analyzed using Statistical Package for Social Sciences (SPSS) version 20 statistical software. Both descriptive and analytic statistics were utilized. The normal distribution of the data was examined using Q-Q plot and histogram. For descriptive analysis, results were expressed as numbers, percentages, and means (±SD). To estimate the potential relationship between patients' TM practice and other variables, Pearson correlation and cross tabulation were used. A *P*-value of < 0.05 was considered statistically significant.

### Operational definition

#### Chronic disease

In this study, a chronic disease, as defined by the U.S. National Center for Health Statistics (US-NCHS), is a disease lasting 1 year or longer (26) and it is a permanent; it leaves residual disability, it is caused by non-reversible pathological alteration, it requires special training of the patient for rehabilitation; or the patient may be expected to require a long period of supervision, observation, or care (15); it includes chronic respiratory diseases, including asthma, COPD, chronic circulatory diseases include HTN, chronic heart failure, chronic endocrine diseases include diabetes, thyroid disorders, chronic musculoskeletal disorders (including rheumatoid diseases), chronic digestive diseases (including pancreatitis), and liver diseases. This study also included the commonest chronic diseases proportionally in the study setting.

## Results

### Sociodemographic characteristics

The study included 384 (91%) of the 422 patients who were approached; other participants (9%) were not involved in the study due to being unable to continue the study. Approximately 232 (60.4%) of the participants were city dwellers. Around one-third (28.4%) of the respondents lacked formal education. Approximately, the monthly income of half (192 or 50.8%) of the participants was between 1,500 and 3,000 Ethiopian birr per month. More than half (214, 55.7%) of the patients used a cellphone, but only around one-third (132, 34.4%) of the participants used a smart phone. Most respondents (371 or 96.6%) used taxis or public transportation to get around. Every 2 months, 169 (44% of the participants) visit the hospital (Table 1).

**Table 1:** Sociodemographic characteristics of chronic disease patients toward telemedicine at ambulatory clinic of UOGCSH, Ethiopia 2022 (*N* = 384).

**Variables**	**Category**	**Frequency (%)**
Sex	Male	205 (53.4)
	Female	179 (46.6)
Age of participants	Mean (±SD)	48.07 (16.17)
	< 31 years	72 (18.8)
	32–50 years	149 (38.8)
	50–60 years	73 (19)
	≥61 years	90 (23.4)
Residence	Urban	232 (60.4)
	Rural	152 (39.6)
Marital status	Single	67 (17.4)
	Married	274 (71.4)
	Divorced	22 (5.7)
	Widow	19 (4.9)
Educational status	No formal education	109 (28.4)
	Primary (1–8)	93 (24.2)
	Secondary (9–12)	104 (27.1)
	College and above	78 (20.)
Occupation	Government employee	60 (15.6)
	Business/self-employee	237 (61.7)
	Unemployed	61 (15.9)
	Freelance	26 (6.8)
Monthly income	≤ 860	28 (7.3)
	861–1,500	37 (9.6)
	1,501–3,000	195 (50.8)
	3,001–4,999	33 (8.6)
	≥5,000	91 (23.7)
Health cost coverage	Out pocket	101 (26.3)
	Health insurance	207 (70.3)
	NGO	7 (1.8)
	Other	6 (1.6)
Internet and /or phone cell use	Yes, on my Owen	214 (55.7)
	On the help of other	18 (4.7)
	No	151 (39.3)
Smart phone use	Yes	132 (34.4)
	My family use it	13 (3.4)
	No	239 (62.2)
Current living situation	Living alone	26 (6.8)
	Living with family	351 (91.4)
	Other	7 (1.8)
Hospital traveling service	Walk	9 (2.3)
	Private car	4 (1)
	Taxi/public transportation	371 (96.6)

### Clinical characteristics of the participants

The most common type of chronic disease among the participants was cardiovascular disorder (84.9%), followed by endocrine disorder (69.0%) and respiratory disorder (65.9%). More than half of the patients (202, 52.6%) had diseases that had lasted <5 years More than half 219 (57%) of the participants had a hospital admission history within the previous 6 months. In the hospital, 143 (37.2%) of participants thought the wait was very long, while 135 (35.2%) thought it was long (Table 2).

**Table 2:** Clinical characteristics of chronic patients at the ambulatory clinic of UOGCSH, Ethiopia 2022 (*N* = 384).

**Variable**	**Chronic disease type**	**Frequency (%)**
Type chronic disease	Cardiovascular disorders	84 (21.9)
	Endocrine disorders	69 (18.0)
	Respiratory disorders	65 (16.9)
	Nerve system disorders	54 (14.1)
	Reproductive disorders	44 (11.5)
	Circulatory disorders	84 (21.9)
	Musculoskeletal disorders	30 (7.8)
	Integumentary disorder	18 (4.7)
	Urinary disorders	17 (4.4)
	Digestive disorders	3 (0.8)
Duration of disease	< 5 years	202 (52.6)
	≥5 years	182 (47.4)
Fallen on the way to our hospital	Yes	61 (15.9)
	No	323 (84.1)
Admission history to the hospital with in 6 months	Yes	219 (57)
	No	165 (43)
Waiting time in the hospital is long	Vey often	143 (37.2)
	Yes	135 (35.2)
	Not so much	53 (13.8)
	No	53 (18.8)
Follow-up time	Monthly	120 (31.3)
	Once every 2 months	169 (44)
	Once every 3 months	79 (20.6)
	Semi-annually	12 (3.1)
	Annually	4 (1)

### Perceptions of participants toward telemedicine

The overall perceptions mean (±SD) score of the respondents was 3.92 (±1.06). About half (189 or 49.2%) of the participants perceived that the TM application was useful for making health decisions for their own health. The majority of the participants (281 or 73.1%) perceived the necessity of TM services for chronic patient care. Similar, the majority of the participants had a positive perception of TM service in terms of faster medical care delivery, saving effort and money, reducing transportation costs, reducing waiting lists in medical care centers, improving communication between the patient and the doctor, and assisting in providing appropriate instructions in emergencies. Overall, close to three-fourths (273 or 71.1%) of the participants had a positive perception of telemedicine services (Table 3).

**Table 3:** Perception toward telemedicine in patients with chronic disease at the UoGCSH, Ethiopia 2022 (*N* = 384).

**Statements**	**Response level**				**Frequency (%)**	
Anxious about using a tablet or mobile device	Very much				81 (21.1)	
	Yes				114 (29.7)	
	Not so much				30 (7.8)	
	No				159 (41.4)	
Opinion about the usefulness of the eHealth application for making decision for own health	Not useful at all				37 (9.6)	
	Not useful				61 (15.9)	
	Unsure				69 (18)	
	Useful				189 (49.2)	
	Very useful				28 (7.3)	
**Perception statements**	**Level of response**, ***n*** **(%)**					
	**Strongly agree**	**Agree**	**Neutral**	**Disagree**	**Strongly disagree**	**Mean (SD)**
Telemedical service may be necessary for chronic patient care	52 (13.5)	229 (59.6)	64 (16.7)	19 (4.9)	20 (5.2)	3.72 (0.94)
Providing a telemedicine service helps faster medical care	62 (16.1)	179 (46.6)	68 (17.2)	19 (4.9)	56 (14.6)	3.74 (1.20)
Providing telemedicine is important for medical care to remote and underserved areas of healthcare.	34 (8.9)	180 (46.9)	104 (27.1)	33 (8.6)	33 (8.6)	3.96 (0.95)
Providing a telemedicine service saves effort.	38 (9.9)	175 (45.6)	105 (27.3)	33 (8.6)	33 (8.6)	3.95 (0.97)
Providing a telemedicine service saves money.	33 (8)	200 (52.1)	85 (22.1)	33 (8.6)	33 (8.6)	3.96 (0.95)
Providing a telemedicine service saves transportation cost.	36 (9.4)	231 (60.1)	45 (11.7)	39 (9.6)	44 (9.8)	3.95 (0.96)
Providing a telemedicine service reduces waiting lists in medical centers.	43 (11.2)	225 (58.6)	52 (13.5)	31 (8.3)	32 (8.5)	3.9 (1.00)
Providing a telemedicine service can improve communication between patients and their doctor or nurse.	62 (16.	179 (46.6)	68 (17.7)	19 (4.9)	56 (14.6)	3.84 (1.120)
Providing a telemedicine service can help in providing appropriate instructions in emergencies.	36 (9.4)	200 (52.1)	54 (14.1)	19 (4.9)	75 (19.5)	4.15 (1.13)
Using eHealth applications to access healthcare offers greater security.	43 (11.2)	201 (52.3)	46 (12)	19 (4.9)	75 (19)	4.15 (1.16)
Easy to share medical information on eHealth applications	39 (10.2)	210 (54.7)	40 (10.4)	20 (5.2)	75 (19.5)	4.15 (1.14)
Using eHealth applications to access healthcare is convenient.	40 (10.4)	195 (50.8)	54 (14.1)	20 (5.2)	75 (19.5)	3.15 (1.15)
Using eHealth application is good to consulting physician	29 (7.6)	200 (52.1)	60 (15.6)	20 (5.2)	75 (19.5)	4.18 (1.10)
eHealth application makes appointment online to see physician	36 (9.4)	216 (56.3)	36 (9.4)	21 (5.5)	75 (19.5)	4.16 (1.13)
Over all mean (± SD) of the respondents						3.92 (1.06)
Overall perception of TM service	Good	273 (71.1%)				
	Poor	111 (28.9%)				

### Perceptions toward privacy issue vs. telemedicine

Around half (48.7%) of the participants strongly believed that they would be worried about the privacy of their health information if it is online, and one quarter (24%) of the respondents strongly perceived that using computers, mobiles, or the internet disclosed their medical information for third person (Table 4).

**Table 4:** Thoughts of participants about using an application to get, keep, and update health information (*N* = 384).

**Statements**	**Level of response**, ***n*** **(%)**				
	**Strongly disagree**	**Disagree**	**Neutral**	**Agree**	**Strongly agree**
I would be worried about the privacy of my health information to use TM	40 (10.4)	35 (9.1)	26 (6.8)	96 (25)	187 (48.7)
I don't need TM to handle my health needs because it does not keep privacy	40 (10.6)	56 (14.6)	57 (14.8)	85 (22.1)	146 (38)
I don't like using computers, mobiles or the internet because it can lead to disclosing medical information to people who are not authorized to do so	96 (24.9)	59 (15.4)	57 (14.8)	80 (20.8)	92 (24)

### Willingness to involve and practice of telemedicine

Around three-fourths (283 or 73.7%) of participants were at least familiar with or had heard about TM. More than one-third 160 (41.7%) of the participants were interested in tracking information about their chronic illness, their diet and calories (146 or 38%), exercise (150 or 39.1%), and being reminded when to take prescriptions (227 or 59.1%). Around half (49.7%) of participants were interested in using the TM website if it came from their doctor, while 216 (56.3%) and 203 (52.9%) were interested in using it if it came from the health insurance plan and the government, respectively. Overall, around two-thirds (63.3%) of participants were willing to participate in the TM service (Table 5).

**Table 5:** Willingness to involve and practice of Telemedicine among chronic patients at the ambulatory clinic of UOGCSH, Ethiopia 2022 (*N* = 384).

**Willingness and practice statement**	**Category**	**Frequency (%)**
Confident toward your own health management	Very confident	87 (22.7)
	Somewhat confident	153 (39.8)
	Not too confident	63 (16.4)
	Not at all confident	81 (21.1)
Are you familiar with telemedicine	Yes	133 (34.6)
	Yes, I have heard, but I don't know the details	150 (39.1)
	No	101 (26.3)
Interest track information about a chronic illness	Yes	160 (41.7)
	No	224 (58.3)
Interest track information to track your diet and calories	Yes	146 (38.0)
	No	238 (62)
Interest information track your exercise	Yes	150 (39.1)
	No	234 (50.9)
Interest information track you remind you when to take prescriptions	Yes	227 (59.1)
	No	157 (40.9)
Interest information track your remind you when you need tests	Yes	175 (2.6)
	No	374 (97.4)
Would you be interested in using this type of website if it were from your doctor	Yes	191 (49.7)
	No	193(50.3)
Would you be interested in using this type of website if it were from the hospital you use	Yes	171 (44.5)
	No	213 (55.5)
Would you be interested in using this type of website if it were from pharmacist	Yes	150 (39.1)
	No	334 (60.9)
Would you be interested in using this type of website if it were from your health insurance plan	Yes	216 (56.3)
	No	168 (43.7)
Would you be interested in using this type of website if it were from a government group like Medicare	Yes	203 (52.9)
	No	184 (47.1)
Would you be interested in using this type of website if it were from nurse	Yes	141 (36.7)
	No	243 (63.3)
Would you be interested in using this type of website if it were from a company like Google or Apple	Yes	138 (35.9)
	No	246 (64.1)
In general, if your health information were online, how worried would you about the privacy and confidentiality of your information?	Very worried	60 (15.6)
	Somewhat worried	142 (37)
	Not too worried	99 (25.8)
	Not at all worried	83 (21.6)
Currently, how much you are interest to use TM application	Very interested	43 (11.2)
	Interested	127 (33.1)
	A little bit interested	73 (19)
	Not at all interested	67 (36.7)
Overall perceived willingness to use TM:	Willing to involve	242 (63.3)
	Not willing to involve	67 (36.7)

### Practice toward telemedicine

**Figure 1:**
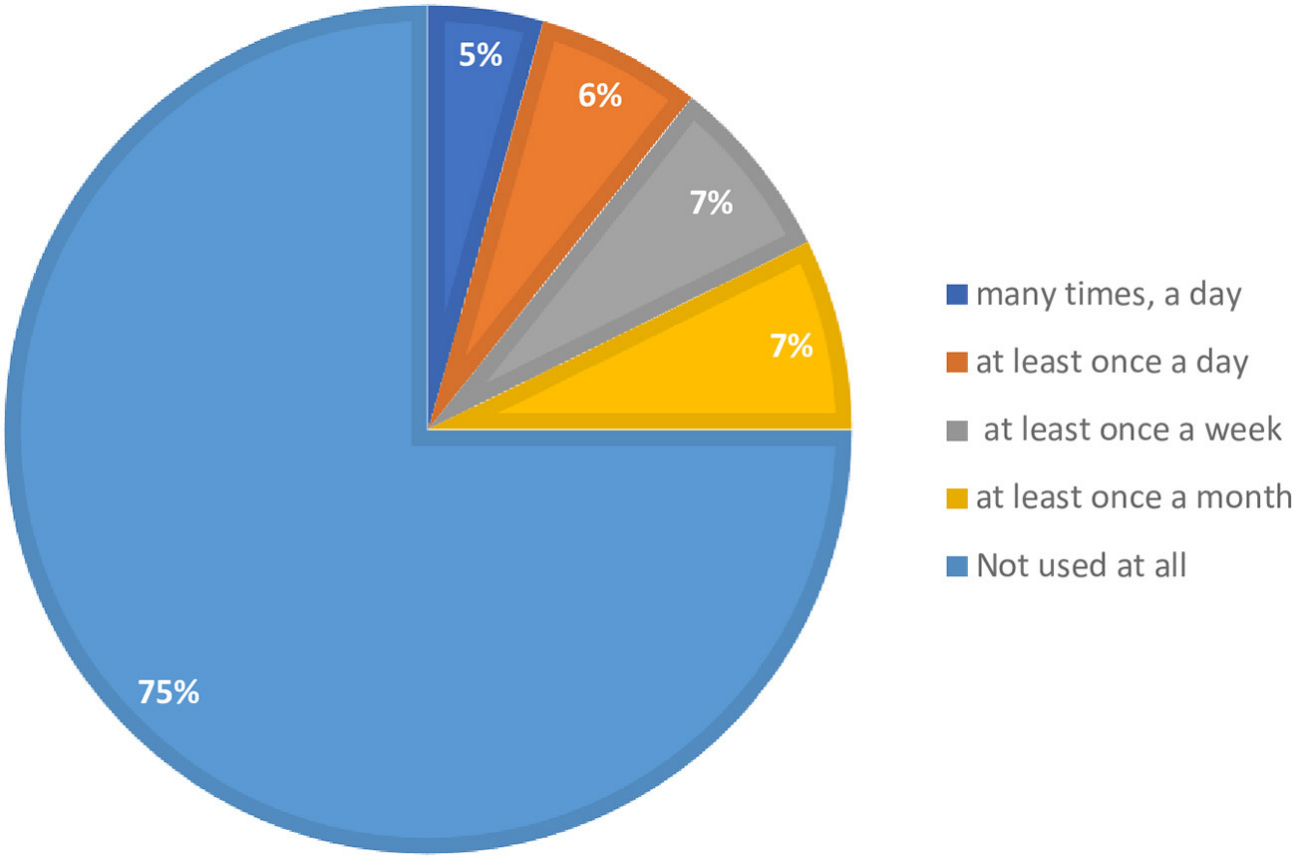
Participants' frequency of using TM applications.

A lower proportion of participants practiced TM for their medical and health-related issues. In addition, three-fourths (75%) of the respondents reported that they did not use the TM application at all for their medical conditions (Figure 1). Overall, only about one-fourth (24.5%) of patients with chronic diseases were perceived to have a high practice of TM application for their medical condition (Table 6).

**Table 6:** Practice of different methods of telemedicine application among chronic diseases patients in ambulatory clinic of UOGCSH, 2022 (*N* = 384).

**Statement**	**Category**	**Frequency (%)**
Have you ever searched online for information about a disease or medical problem	Yes	31 (8.9)
	No	350 (91.1)
Have you ever searched online for information about a doctor	Yes	16 (4.2)
	No	368 (95.8)
Have you ever typed information on an application about your dietary, physical exercise, and overall lifestyle modifications	Yes	16 (4.2)
	No	368 (95.8)
Have you ever typed information on an application about a chronic illness you have	Yes	18 (4.7)
	No	366 (95.3)
Have you ever renewed a prescription online	Yes	13 (3.4)
	No	371 (96.6)
Have you ever consulted your doctor using TM	Yes	11 (2.9)
	No	373 (97.1)
Have you ever used a personal health record for your health	Yes	7 (1.8)
	No	377 (98.2)
Have you ever looked at a test result online	Yes	13 (3.4)
	No	371 (96.6)
Have you ever used a device that measures health information (like blood pressure; blood glucose levels) that connects to your mobile/website application	Yes	5 (1.3)
	No	379 (98.7)
Have you ever posted anything online about your health or health care	Yes	5 (1.3)
	No	379 (98.7)
Have you ever Joined an online group that is for a health issue that you or your family member has	Yes	13 (3.4)
	No	371 (96.6)
Have you ever booked appointment with doctors using TM	Yes	29 (7.6)
	No	355 (92.4)
Overall perceived level of TM practice	High	94 (24.5)
	Low	290 (75.5)

### Association between patient demographic character and inclination to use TM

Cell phone or internet use, use of a smartphone, healthcare insurance coverage, anxious to use tablet or device, interest to use eHealth application, frequency of using health application, and frequency of follow-up time had a statistically significant association with TM practice (Table 7).

**Table 7:** Comparison of patients' background characteristics between patients in terms of practicing TM.

**Characteristics**	**Category**	**TM practice**		***P*-value**
		**High**	**Low**	
Age	Mean (± SD)	46.4 (17.2)	48.58 (15.7)	0.12
Sex	Male	57 (27.8)	148 (72.2)	0.154
	Female	37 (20.7)	142 (79.3)	
Use of internet or cell phone	Yes, on my own	49 (22.9)	165 (77.1)	0.006^*^
	Yes, with the help of others	10 (55.6)	8 (44.4)	
	No	33 (21.9)	118 (78.1)	
Use of smart phone	Yes	38 (28.8)	94 (71.2)	0.001^*^
	My family use it	9 (69.2)	4 (30.8)	
	No	45 (18.8)	194 (81.2)	
Health coverage	Payment	31 (30.7)	70 (69.3)	0.007^*^
	Insurance	54 (20)	216 (80)	
	NGO	5 (42.3)	8 (57.8)	
Living condition	Living alone	10 (38.5)	16 (61.5)	0.092
	Living with family	79 (22.5)	272 (77.5)	
	Other	3 (42.9)	4 (57.1)	
Admission to the hospital	Yes	48 (21.9)	171 (78.1)	0.118
	No	46 (27.9)	119 (72.1)	
Fall in the way to hospital	Yes	13 (21.3)	48 (78.7)	0.237
	No	81 (25.1)	242 (74.9)	
Anxious to use tablet or device	Very much	14 (17.3)	67 (82.7)	0.021^*^
	Yes	24 (21.1)	90 (78.9)	
	No so much	12 (40)	18 (60)	
	No	44 (27.7)	115 (72.3)	
Interest to use eHealth application for your health information	Very interested	19 (41.5)	24 (58.5)	0.033^*^
	Somewhat interested	32 (24.8)	97 (75.2)	
	Not too interested	14 (19.2)	59 (80.8)	
	Not all interested	29 (20.6)	112 (79.4)	
Frequency of using health application	Many times, a day	10 (58.8)	7 (41.2)	< 0.001^*^
	At least once a day	9 (37.5)	15 (62.5)	
	At least once a week	17 (63)	10 (37)	
	At least once a month	17 (60.7)	11(39.3)	
	Not used at all	39 (13.5)	249 (86.5)	
Frequency of follow-up time	Monthly	20 (17.7)	100 (82.3)	< 0.001^*^
	Once every 2 months	37 (21.9)	132 (78.1)	
	Every 3 month	28 (35.4)	51 (64.6)	
	Semi annually	3 (25)	9 (75)	
	Annually	3 (75)	1 (25)	

* Indicates variables with P < 0.05.

## Discussion

The current study sought to determine the level of perception, willingness, and extent of TM practice among patients with chronic diseases. This study showed that most participants had a positive perception of and willingness to use TM services. However, their perceived practice level was low. The study's main finding is that familiarity with TM influences all attitudes, willingness, and practice of TM.

The current study showed that around three-fourths of participants had a positive perception of the TM service. This finding is similar to that of studies conducted in Saudi Arabia (27) and Bangladesh (28), which found that the majority of patients had a positive attitude toward TM. However, the current finding is contrary to a study in the Netherlands (14), which showed that the majority of respondents had a negative attitude toward the TM service. The discrepancy might be because of higher awareness regarding TM services use in the current time, especially related to COVID-19 epidemic, but the Netherlands' study was conducted before the epidemic, and the impact of TM may not have been sufficiently recognized earlier. The other possible reason might be that the eHealth implementation in the earlier study was collaborative with patients and carers. This may result in a lower attitude because of the service costs. Furthermore, technology-based applications might also be a reason for higher perception.

The majority of participants were willing to participate in the TM service. This finding is in line with earlier studies across the world regarding patients' willingness to use digital-based health services (26, 28–30). A previous study among diabetes patients in Ethiopia showed that more than two-thirds of participants were inclined to use mobile-based health services (30). A study conducted in Australia also revealed that the majority of participants were satisfied and willing to utilize TM if it reduced costs and waiting times (29). In addition, another study conducted in Pakistan showed that more than half of the participants were willing to rely on text messages to communicate with healthcare providers (31). Furthermore, according to a study conducted in Bangladesh, a high proportion of participants were eager to learn about TM. They believed that TM could benefit the ongoing advancement of healthcare (28). A Cochrane review also showed that a digital-based text message intervention improved patients' self-management practices and made patients more involved in the TM service (23). These findings may suggest that digital-based health interventions could be popular and advancement in the healthcare system from the patient's perspective.

However, in terms of TM practice, this study revealed that around three-fourths of the participants had overall low TM practice. This finding is consistent with a study conducted in the Netherlands, which found that most applications were practiced low (14). On the contrary, a study in Poland found that the majority of participants had used TM in various ways (32). In addition, according to an Australian study, close to half of the respondents used TM applications (29). Furthermore, studies in Pakistan (31) and Saudi Arabia (27) also showed that a higher proportion of respondents utilized TM services in different ways. The discrepancy could be because of differences in digital-enabled infrastructure and healthcare systems across settings. Internet access, socioeconomic status, and TM implementation differences might affect the application of the service among chronic disease patients. The other possible difference might be awareness of privacy issues and digital-related health applications. In this study, most participants worried and believed that the digital-based service jeopardized their privacy. This study suggests that patients can be attracted to a digital-based system by increasing awareness, promoting pushing factors and encouraging driving factors. The study revealed that the use of the internet, having a smartphone, and interest in and frequency of using health applications were among the significant factors associated with the practice of TM services among patients. This is because their experience with the practices and techniques helps them engage easily. As a result, better opportunities could be provided to fully implement the TM service in Ethiopia.

### Implication of this study

Assessing the perception, willingness, and practice of long-term patients in TM implementation has a paramount role in establishing and implementing TM. Identifying barriers to its implementation is an initial step to providing possible solutions for better control of chronic diseases through TM. There is substantial evidence to support the benefits of TM in the control of chronic diseases and the general advancement of the health care delivery system. This study focused on assessing chronic disease patients' perception, willingness, and practice toward telemedicine. A well-developed perception, willingness, and practice of TM will provide a baseline for the identification of barriers and fill the gaps to ensure better health care through TM implementation. This will help design digital-based interventions for managing chronic disease patients. Furthermore, it will extend a body of knowledge to patients, carers, practitioners, the community, stakeholders, and the healthcare system community in general. The findings of this study will also serve as a baseline for future research in the area. In addition, these findings will also help to scale up and integrate with other digital health policies and initiatives.

### Strengths and limitation of the study

The results of this study should be interpreted while remembering the following limitations: First, due to the nature of the study design, a cause-and-effect relationship could not be explained. The finding relies on self-reported outcomes from participants, which could depend on their faith and honesty. In addition, the sample size calculation did not take into account the prevalence of chronic diseases in Northwest Ethiopia. This may affect the sample size calculation results. Despite the limitations, our findings highlighted the attitude, willingness, and extent of TM practice among patients with chronic disease. It could be a baseline for future research in this area. In the future, it would be welcome to explore the opportunities and challenges of practicing a digital-based healthcare system in Ethiopia using a prospective national-based study.

## Conclusion

This study concluded that although patients with chronic diseases perceived and willingly accepted TM services, their perceived practice level was low. Experience with health applications and the availability of the internet and cell phones were among the factors associated with practicing TM. In general, this study provides baseline information about perceptions, willingness, and actual practice of digital-based healthcare among Ethiopian chronic disease patients. Creating awareness of privacy issues vs. digital-based health applications and creating an opportunity may result in practicing the available digital health system among patients and even for an entire population.

## Data availability statement

The raw data supporting the conclusions of this article will be made available by the authors, without undue reservation.

## Ethics statement

Research proposal was reviewed and approved by the Research and Ethics Review Committees of the School of Pharmacy at the University of Gondar with a reference number of UOG-SOP-257/2022. The patients/participants provided their written informed consent to participate in this study.

## Author contributions

EAB and AKS participated in the conception, design, administration, and supervising of the study, while DG, AKN, and EG contributed in the data curation, methodology, and interpreted the data. All authors drafted the initial manuscript, read, approved the final manuscript, and contributed the critical review and the content.

## References

[1] Blandford A Wesson J Amalberti R AlHazme R Allwihan R. Opportunities and challenges for telehealth within, and beyond, a pandemic. Lancet Glob Health. (2020) 8:e1364–5. 10.1016/S2214-109X(20)30362-4 32791119 PMC7417162

[2] Portnoy J Waller M Elliott T. Telemedicine in the era of COVID-19. J Allergy Clin Immunol Pract. (2020) 8:1489–91. 10.1016/j.jaip.2020.03.008 32220575 PMC7104202

[3] Novara G Checcucci E Crestani A Abrate A Esperto F Pavan N. Telehealth in urology: a systematic review of the literature. How much can telemedicine be useful during and after the COVID19 pandemic? Eur Urol. (2020) 78:786–811. 10.1016/j.eururo.2020.06.025 32616405 PMC7301090

[4] Sim I. Mobile devices and health. N Engl J Med. (2019) 381:956–68. 10.1056/NEJMra1806949 31483966

[5] Almathami HKY Win KT Vlahu-Gjorgievska E. Barriers and facilitators that influence telemedicine-based, real-time, online consultation at patients' homes: systematic literature review. J Med Int Res. (2020) 22:e16407. 10.2196/16407 32130131 PMC7059083

[6] Calim S Ulaş SC Demirci H Tayhan EB. Effects of high fidelity simulation model on midwives' shoulder dystocia management skills: an educational Intervention study. Nigerian J Clin Pract. (2022) 25:773. 10.4103/njcp.njcp_1393_21 35708417

[7] Breen G-M Matusitz J. An evolutionary examination of telemedicine: a health and computer-mediated communication perspective. Soc Work Public Health. (2010) 25:59–71. 10.1080/19371910902911206 20300559 PMC2838709

[8] Gu D Humbatova G Xie Y Yang X Zolotarev O Zhang G. Different roles of telehealth and telemedicine on medical tourism: an empirical study from Azerbaijan. InHealthcare. (2021) 9:1073. 10.3390/healthcare9081073 34442210 PMC8392188

[9] Lustig TA. The Role of Telehealth in an Evolving Health Care Environment: Workshop Summary. Washington, DC: National Academies Press (2012). Available online at: https://nap.nationalacademies.org/catalog/13466/the-role-of-telehealth-in-an-evolving-health-care-environment 24901186

[10] Assaye BT worku Shimie A. Telemedicine use during COVID-19 pandemics and associated factors among health professionals working in health facilities at resource-limited setting 2021. Inform Med Unlocked. (2022) 33:101085. 10.1016/j.imu.2022.101085 36105540 PMC9462923

[11] Bokolo AJ. Exploring the adoption of telemedicine and virtual software for care of outpatients during and after COVID-19 pandemic. Irish J Med Sci. (2021) 190:1–10. 10.1007/s11845-020-02299-z 32642981 PMC7340859

[12] Chitungo I Mhango M Mbunge E Dzobo M Musuka G Dzinamarira T. Utility of telemedicine in sub-Saharan Africa during the COVID-19 pandemic. A rapid review. Hum Behav Emerg Technol. (2021) 3:843–53. 10.1002/hbe2.297 34901772 PMC8653215

[13] Jnr BA. Use of telemedicine and virtual care for remote treatment in response to COVID-19 pandemic. J Med Syst. (2020) 44:132. 10.1007/s10916-020-01596-5 32542571 PMC7294764

[14] Hofstede J De Bie J Van Wijngaarden B Heijmans M. Knowledge, use and attitude toward eHealth among patients with chronic lung diseases. Int J Med Inform. (2014) 83:967–74. 10.1016/j.ijmedinf.2014.08.011 25269992

[15] Shirwar AK Bharati AS. An ayurvedic management of kitibha kushta (plaque psoriasis)-a case study. J Ayurveda Integr Med Sci. (2022) 7:194–9. Available online at: https://jaims.in/jaims/article/view/1874

[16] Yosef T. Prevalence and associated factors of chronic non-communicable diseases among cross-country truck drivers in Ethiopia. BMC Public Health. (2020) 20:7. 10.1186/s12889-020-09646-w 33069207 PMC7568414

[17] Tesfay FH Zorbas C Alston L Backholer K Bowe SJ Bennett CM. Prevalence of chronic non-communicable diseases in Ethiopia: a systematic review and meta-analysis of evidence. J Frontiers in Public Health. (2022) 10:936482. 10.3389/fpubh.2022.936482 35991039 PMC9385028

[18] Haleem A Javaid M Singh RP Suman R. Telemedicine for healthcare: capabilities, features, barriers, and applications. Sens Int. (2021) 2:100117. 10.1016/j.sintl.2021.100117 34806053 PMC8590973

[19] Fraiche AM Eapen ZJ McClellan MB. Moving beyond the walls of the clinic: opportunities and challenges to the future of telehealth in heart failure. JACC Heart Fail. (2017) 5:297–304. 10.1016/j.jchf.2016.11.013 28189579

[20] Rachas A Farmer AJ Inzitari M Shepperd S. Interactive telemedicine: effects on professional practice and health care outcomes. Cochrane Database Syst Rev. (2015) 9:CD002098. 10.1002/14651858.CD002098.pub2 26343551 PMC6473731

[21] Mao Y Lin W Wen J Chen G. Impact and efficacy of mobile health intervention in the management of diabetes and hypertension: a systematic review and meta-analysis. BMJ Open Diab Res Care. (2020) 8:e001225. 10.1136/bmjdrc-2020-001225 32988849 PMC7523197

[22] Alboraie M Allam MA Youssef N Abdalgaber M El-Raey F Abdeen N. Knowledge, applicability, and barriers of telemedicine in Egypt: a national survey. Int J Telemed Appl. (2021) 2021:5565652. 10.1155/2021/5565652 34211550 PMC8192215

[23] Shi C. Mobile phone messaging for facilitating self-management of long-term illnesses: summaries of nursing care-related systematic reviews from the cochrane library. Int J Evid Based Healthc. (2013) 11:344–5. 10.1111/1744-1609.12041

[24] Kola L Kohrt BA Hanlon C Naslund JA Sikander S Balaji M. COVID-19 mental health impact and responses in low-income and middle-income countries: reimagining global mental health. Lancet Psychiatry. (2021) 8:535–50. 10.1016/S2215-0366(21)00025-0 33639109 PMC9764935

[25] Manyazewal T Woldeamanuel Y Blumberg HM Fekadu A Marconi VC. The potential use of digital health technologies in the African context: a systematic review of evidence from Ethiopia. NPJ Digital Med. (2021) 4:125. 10.1038/s41746-021-00487-4 34404895 PMC8371011

[26] Bernell S Howard SW. Use your words carefully: what is a chronic disease? Front Public Health. (2016) 4:159. 10.3389/fpubh.2016.00159 27532034 PMC4969287

[27] Alajwari HA Alfayez A Alsalman D Alanezi F Alhodaib H Al-Rayes S. Knowledge and attitude of Saudi Arabian citizens towards telemedicine during the COVID-19 pandemic. Int Health. (2022) 14:604–9. 10.1093/inthealth/ihab082 34893850 PMC8689698

[28] Haque MMA Jahan Y Khair Z Moriyama M Rahman MM Sarker MH. Perceptions about Telemedicine among Populations with Chronic Diseases amid COVID-19: data from a Cross-Sectional Survey. Int J Environ Res Public Health. (2022) 19:4250. 10.3390/ijerph19074250 35409932 PMC8998658

[29] Shiferaw F Zolfo M. The role of information communication technology (ICT) towards universal health coverage: the first steps of a telemedicine project in Ethiopia. Glob Health Action. (2012) 5:15638. 10.3402/gha.v5i0.15638 22479235 PMC3318899

[30] Jemere AT Yeneneh YE Tilahun B Fritz F Alemu S Kebede M. Access to mobile phone and willingness to receive mHealth services among patients with diabetes in Northwest Ethiopia: a cross-sectional study. BMJ Open. (2019) 9:e021766. 10.1136/bmjopen-2018-021766 30679284 PMC6347931

[31] Iftikhar S Saqib A Sarwar MR Sarfraz M Arafat M Shoaib Q-u-a. Capacity and willingness to use information technology for managing chronic diseases among patients: a cross-sectional study in Lahore, Pakistan. PLoS ONE. (2019) 14:e0209654. 10.1371/journal.pone.0209654 30629632 PMC6328230

[32] Prevett M. Chronic non-communicable diseases in ethiopia-a hidden burden. Ethiop J Health Sci. (2012) 22(S):1–2. Available online at: https://www.ncbi.nlm.nih.gov/pmc/articles/PMC3542741/23319834 PMC3542741

